# Linear and non-linear properties of feature selectivity in V4 neurons

**DOI:** 10.3389/fnsys.2015.00082

**Published:** 2015-05-27

**Authors:** Jon Touryan, James A. Mazer

**Affiliations:** ^1^Department of Neurobiology, Yale School of MedicineNew Haven, CT, USA; ^2^Human Research and Engineering Directorate, U.S. Army Research LaboratoryAberdeen, MD, USA; ^3^Department of Psychology, Yale UniversityNew Haven, CT, USA

**Keywords:** feature selectivity, extra-striate visual cortex, receptive field, natural scenes, Gaussian bubbles

## Abstract

Extrastriate area V4 is a critical component of visual form processing in both humans and non-human primates. Previous studies have shown that the tuning properties of V4 neurons demonstrate an intermediate level of complexity that lies between the narrow band orientation and spatial frequency tuning of neurons in primary visual cortex and the highly complex object selectivity seen in inferotemporal neurons. However, the nature of feature selectivity within this cortical area is not well understood, especially in the context of natural stimuli. Specifically, little is known about how the tuning properties of V4 neurons, measured in isolation, translate to feature selectivity within natural scenes. In this study, we assessed the degree to which preferences for natural image components can readily be inferred from classical orientation and spatial frequency tuning functions. Using a psychophysically-inspired method we isolated and identified the specific visual “driving features” occurring in natural scene photographs that reliably elicited spiking activity from single V4 neurons. We then compared the measured driving features to those predicted based on the spectral receptive field (SRF), estimated from responses to narrowband sinusoidal grating stimuli. This approach provided a quantitative framework for assessing the degree to which linear feature selectivity was preserved during natural vision. First, we found evidence of both spectrally and spatially tuned suppression within the receptive field, neither of which were present in the linear SRF. Second, we found driving features that were stable during translation of the image across the receptive field (due to small fixational eye movements). The degree of translation invariance fell along a continuum, with some cells showing nearly complete invariance across the receptive field and others exhibiting little to no position invariance. This form of limited translation invariance could indicate that a subset of V4 neurons are insensitive to small fixational eye movements, supporting perceptual stability during natural vision.

## Introduction

A fundamental challenge in visual neuroscience is to understand and model the relationship between arbitrary complex visual stimuli and the corresponding patterns of activity they evoke in visual neurons. There has been an ongoing debate about whether or not the stimulus-response relationship measured in visual neurons is dependent on the class of visual stimuli used or method taken to characterize the relationship. Specifically, some have questioned whether the visual features that drive neuronal activity during natural vision are the same features that allow synthetic or “unnatural” stimuli to drive neurons under laboratory conditions (Felsen and Dan, 2005; Rust and Movshon, 2005). In primary visual cortex (V1), the spectral receptive field (SRF) provides a compact representation of the linear component of neuronal selectivity for stimulus orientation, spatial frequency and often spatial phase (Deangelis et al., 1993; Wu et al., 2006). In V1 SRFs are frequently estimated from responses to narrowband or so-called “simple” stimuli, like sinusoidal gratings (Ringach et al., 1997; Mazer et al., 2002) (although see David et al. (2004) and Touryan et al. (2005) for examples of estimating SRFs from responses to spectrally complex stimuli). Despite the fact that V1 neurons exhibit a number of well-established static and dynamic non-linearities [e.g., spiking thresholds (Chichilnisky, 2001) and contrast gain control (Ohzawa et al., 1982; Heeger, 1992)], in many instances the linear SRF accurately predicts responses to both narrowband (i.e., sinusoidal gratings) and more complex natural scene stimuli (Theunissen et al., 2001; David et al., 2004). Importantly, if the SRF reflects an independent model of feature selectivity, then it should be able to predict responses to any stimuli, natural or unnatural, with a reasonable degree of accuracy. Consistent with this, Felsen et al. (2005) demonstrated that in V1 the SRF, even when computed from responses to simple stimuli, can be used to readily identify key features in natural scenes that drive neurons to spike.

This universality is notably absent in area V4, where SRFs estimated from responses to narrowband stimuli generally fail to predict responses to broadband or natural stimuli (David et al., 2006; Oleskiw et al., 2014), a strong indicator that non-linear mechanisms substantially influence responses. Many studies have used carefully designed broadband, but not necessarily natural, stimuli to demonstrate that V4 encodes a substantial amount of information about complex 2D image properties. These include stimulus shape (Desimone and Schein, 1987; Gallant et al., 1993; Kobatake and Tanaka, 1994; Pasupathy and Connor, 2001, 2002), color (Zeki, 1980), texture (Hanazawa and Komatsu, 2001) and disparity (Hinkle and Connor, 2001, 2002). However, it is unclear how linear or quasi-linear tuning for isolated stimulus properties (e.g., tuning curves or surfaces for orientation, spatial frequency or contour curvature) can be generalized to predict the response of neurons to more complex shapes or objects that occur within the context of natural scenes. Importantly, while there is broad agreement that linear SRF models fail to predict responses to complex stimuli in V4, the reasons for these failures are not yet well understood. Likewise, there is currently no model of V4 feature selectivity that can be universally applied to all classes of stimuli; that is, a model that can accurately predict responses to stimuli of arbitrary complexity independent of the stimulus set used to construct the model.

To address these issues, we adapted a psychophysical masking technique known as “bubbles” (Gosselin and Schyns, 2001) to identify and characterize the non-linear shape or feature tuning properties of V4 neurons. Bubble-masking has been previously used in conjunction with neurophysiological methods to relate the visual selectivity of inferotemporal neurons to the features used by human and monkey observers to discriminate complex images (Nielsen et al., 2006, 2008). The approach taken in the present study was specifically designed to identify non-linearities active during natural vision that are related to the neural encoding of spectrally complex stimuli in the early stages of extrastriate processing. Specifically, we recorded neuronal responses to repeated presentations of natural scene stimuli partially masked at random locations during each presentation. We used sets of spatially localized, transparent Gaussian windows to identify the neuronal “driving features,” corresponding to the minimum set of pixels that reliably drives the neural response, for each image. We then compared the spatial and spectral properties of measured driving features to those predicted by the linear SRF, which was estimated from responses to a dynamic sinusoidal luminance grating sequence (Ringach et al., 1997; Mazer et al., 2002). Mismatches between measured and predicted driving features reflect inherent non-linearities in each neuron's tuning function. The specific pattern of mismatches, or failures of the linear model, allowed us to garner new insights into how V4 circuits contribute to shape selectivity.

## Materials and methods

### Data collection

Data were collected from two adult male monkeys (*Macaca mulatta*), 10–12 kg. All procedures were in accordance with the NIH Guide for the Care and Use of Laboratory Animals and approved by the Yale University Institutional Animal Care and Use Committee. In two separate sterile surgeries performed under isoflurane anesthesia, a Titanium headpost (AZ Machining, Boston, MA) and acrylic recording platform (Dentsply, Milford, DE) were affixed to the skull using bone screws (Synthes, West Chester, PA). Following acclimation to head restraint and subsequent behavioral training on a fixation task (see below), a stainless steel 5 mm recording chamber was attached to the platform directly over V4 and a burr-hole craniotomy was performed under Ketamine (10 mg/kg) and Midazolam (0.1 mg/kg) anesthesia to provide microelectrode access. V4 was targeted using stereotaxic coordinates and skull morphology and subsequently confirmed based on physiological properties of recorded cells (i.e., neuronal response latency, receptive field size and visual field eccentricity [see Supplementary Material]).

Task timing, stimulus presentation and data collection were controlled by a Linux PC running pype (https://github.com/mazerj/pype3). Stimuli were presented on a gamma corrected (linearized) Viewsonic G810 CRT display with an 85 Hz frame rate and a resolution of 1025 × 768 pixels (39 × 29 cm) viewed at a distance of 66 cm. Eye movements were recorded digitally at a minimum of 500 Hz using an infrared eye tracker (EyeLink 1000, SR Research, Toronto, Canada), and single neuron activity was recorded with high impedance (nominally 10–25 MΩ) epoxy coated tungsten microelectrodes (125–200 μm diameter, 20–25 degree taper; Frederick Haer Co., New Brunswick, ME). One to two electrodes at a time were advanced transdurally with a motorized microdrive system (Graymatter Research, Bozeman, MT). Neural signals were amplified, filtered and discriminated (MAP, Plexon Inc., Dallas, TX) and spike times recorded with 1 ms precision.

### Fixation task and receptive field characterization

Animals were trained to fixate on a 2–3 arcmin 100% contrast square fixation target for up to 3 s (1° radius window). After a random time interval (truncated exponential distribution), the fixation target dimmed and animals had to either contact or release a touch bar within 300 ms to obtain a liquid reward. Following fixation breaks, premature (<70 ms) and late touch bar responses, the display was briefly flashed red to indicate an error, followed by a 1–2 s timeout period. During periods of fixation, high contrast black and white probe stimuli were flashed in randomized order on an invisible grid at 5–10 Hz to map the spatial RF of each neuron studied. Preliminary hand mapping was used to set probe orientation and grid position. RF location and size (radius) were determined by fitting the half-maximal iso-response contour of the spike-triggered average with a circle (Jones and Palmer, 1987; Mazer et al., 2002).

Each neuron's linear feature selectivity was estimated from responses to a dynamic sequence of sinusoidal gratings presented at 10 Hz centered in the RF. Grating orientation, spatial frequency and spatial phase (Ringach et al., 1997; Mazer et al., 2002) were selected at random for each 100 ms stimulus frame. Gratings were sized to fill the classical RF (CRF) and smoothly alpha-blended into the uniform gray screen background, using a trapezoidal envelope function, to avoid high spatial frequency transients at the stimulus boundary. SRFs were estimated by computing the parametric spike-triggered average stimulus in the spatial frequency domain using a fixed latency of 50 ms and a 100 ms integration window. These values were selected to capture the complete response period for all neurons included in the study based on an exploratory analysis of impulse response functions in our data. Consistent with previous reports, we found little evidence of tuning for absolute spatial phase in V4 neurons (David et al., 2006) and therefore collapsed SRFs across phase for all subsequent analyses.

### Bubble-masked natural scenes

**Figure 1:**
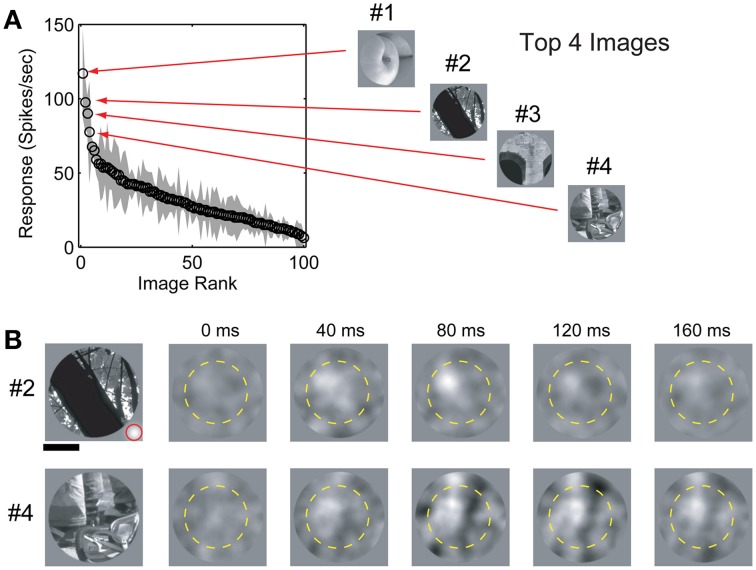
**Image selection and spike-triggered masks. (A)** Response (mean ± std) of a single V4 neuron to a set of 100 randomly selected natural scene images sorted by firing rate. #1–#4 indicates the four “top” (maximal evoked response) images. Spike-triggered masks were estimated for the top 4 images. **(B)** Spike-triggered masks from the same cell for images #2 and #4 in **(A)**. The dashed yellow circle indicates the RF position and size (see text for details). Scale bar indicates 0.5 degree and the size of an individual Gaussian window is indicated (red circle) in the lower right corner of image #2.

**Figure 2:**
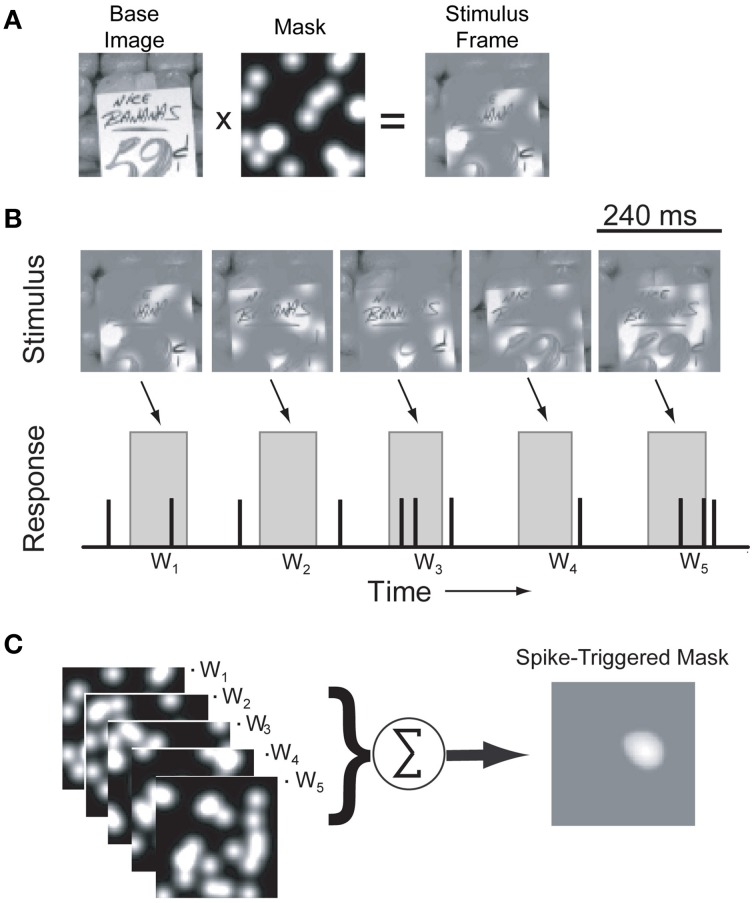
**Identification of driving features using bubble-masked natural images. (A)** Each frame of the stimulus sequence was created by combining a pre-selected natural scene photograph (see Materials and Methods) with an opaque mask perforated by randomly distributed Gaussian windows. **(B)** Example stimulus frames for a single image and a simulated response. In each frame, the positions of the Gaussian windows are randomized. During the actual experiment, underlying base images were randomly interleaved to minimize adaptation effects (see text for details). Stimuli were presented at 4.25 Hz (240 ms/frame) and responses to each stimuli (*w_n_*) were determined by computing the mean firing rate in a window 50–150 ms after stimulus onset (gray boxes). **(C)** For each underlying image, masks were weighted by the recorded neuronal response and averaged to estimate an image-specific spike-triggered mask.

Identification of the neuronal driving features was a two-step process illustrated in Figures 1–2. We first identified a small number of vignetted natural scene photographs (black and white) that robustly elicited neuronal firing by presenting a sequence of 100 randomly selected natural scene stimuli at 4.25 Hz, centered in the RF (Figure 1A). Although many V4 neurons are color-selective (Zeki, 1980), Bushnell and Pasupathy (2012) recently demonstrated that form selectivity in V4 is color invariant; therefore we used only black and white images in this study to maximize the number of stimulus repetitions for each neuron. The set of images was presented in random order 4–10 times for each cell. As with the sinusoidal gratings, each image was scaled or cropped to fill the CRF of the neuron being studied and smoothly blended into the gray background. We then identified 2–6 images that evoked the highest average firing rates. Subsequently, these “top” images were randomly masked with bubbles and presented in a continuous stream centered in the RF at 4.25 Hz while monkeys maintained fixation. Each opaque bubble mask covered the entire underlying image and contained 20 transparent Gaussian windows (σ = 7 pixels) distributed at randomized locations throughout the mask. The positions of all 20 windows were selected at random from a uniform distribution on each frame of the stimulus sequence (Figure 2B). Each Gaussian window revealed a different portion of the underlying image; on average, 32% of the image was visible on any given frame. The selection of this window size and density was determined from a pilot study (data not shown) and represents a balance between resolution, mapping time, and the average effectiveness of the masked images to elicit a response. The 2–6 most effective natural images were randomly interleaved on each frame to minimize neuronal adaptation effects and to reduce the likelihood of perceptual completion or filling in, which could result in non-stationary neuronal response dynamics.

### Driving feature identification

Responses to bubble masked images were analyzed off-line using custom MATLAB (MathWorks, Natick, MA) functions. For each of the top images used, mask patterns (ignoring the underlying image pixels) were weighted by the evoked response and averaged to calculate the spike-triggered mask (Chichilnisky, 2001; Touryan et al., 2002). Initially, the evoked responses were averaged and spike-triggered masks calculated in a sequence of 40 ms bins from stimulus onset (Figure 1B). To improve the statistical power of the spike-triggered masking technique we subsequently limited our calculation of the evoked response to a single window between 50 and 150 ms after stimulus onset, which captured the majority of the response dynamics observed in the initial analysis (see below). These spike-triggered masks isolate image regions in each stimulus that effectively modulate neuronal firing. Bootstrapping and reshuffling methods (Efron and Tibshirani, 1993) were used to assess the statistical significance of each mask pixel: for each cell, we generated a null distribution of spike-triggered masks by shuffling spike rates across the ensemble of mask stimuli. We then calculated 99.5% confidence intervals on the null distribution and set pixels in the measured spike-triggered mask to zero if they fell within that confidence interval. The resulting spike-triggered mask represents the weighted contribution of each image pixel to the neuronal firing rate for a given base image (Figure 2).

Although the Gaussian window positions were independent and uncorrelated, the windows themselves have local spatial correlation structure (i.e., adjacent mask positions tend to have similar values). This accounts for the visible smoothness in the spike-triggered masks even though these masks were never smoothed. Importantly, since the spike-triggered masks in this study were used solely to identify and characterize image regions that elicit a response, as opposed to measuring fine-grained feature selectivity, we made no attempt at spatial de-correlation. One consequence of this approach is that our spike-triggered masks represent an upper-bound on the pixels required to elicit responses. The degree to which spike-triggered masks could overestimate the number of driving pixels is dictated by the size of the individual bubbles. In our stimuli, the bubbles were relatively small (area < 150 pixels; see Figure 1B) compared to the size of the estimated masks (mean mask area = 733 ± 607 pixels; all values are mean ± STD unless otherwise noted). As noted above, the bubble size represents an empirically derived compromise for maximizing spatial resolution while minimizing recording time. On average, the area of the estimated masks was less than 6% of the underlying 128 × 128 pixel base images.

### Linear model

**Figure 3:**
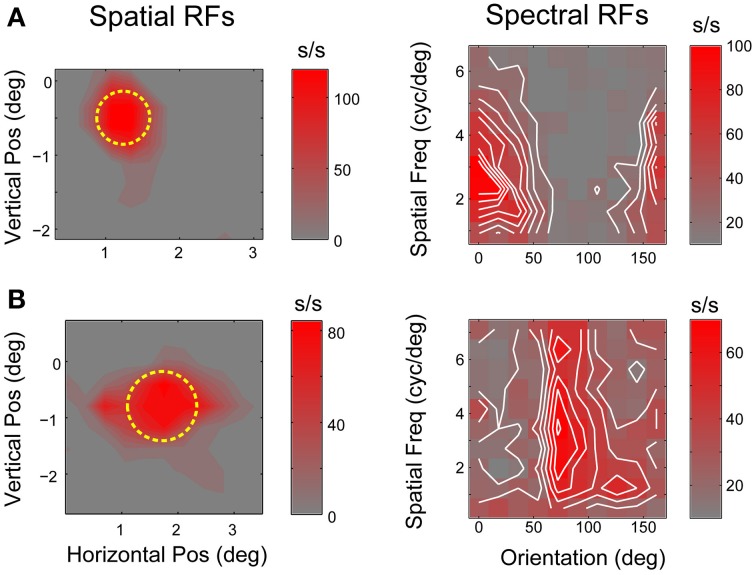
**V4 linear receptive fields. (A,B)** Spatial and spectral receptive fields for two typical V4 neurons. Left panels show spatial RFs estimated by reverse correlation of responses to black and white probe stimuli flashed on a randomized grid in and around the receptive field. Yellow circles indicate RF location and extent (see text for details). Right panels show SRFs estimated from responses to sinusoidal flashed grating stimuli. Each plot shows the joint orientation-spatial frequency tuning of a single neuron. Iso-response contours (white lines) are separated by intervals of 10 spikes/s.

Each neuron's spatial and spectral RFs (Figure 3), derived from responses to flashed bars and gratings, were used to predict the excitatory and suppressive features in the natural image stimuli using a quasi-linear model (David et al., 2004, 2006). The phase-collapsed SRF used here incorporates a single, explicit non-linearity similar to the phase invariance found in striate cortex complex cells (Touryan et al., 2002). For each neuron, the SRF was used to construct a Fourier domain, amplitude-only filter (i.e., no spatial phase selectivity) corresponding to the joint orientation-spatial frequency tuning matrix (Mazer et al., 2002). Filters were normalized to produce a unity response to the optimal grating stimulus and applied to the top natural images for each neuron (Figure 1A). Thus, only image components with orientations and spatial frequencies within the neuron's passband were preserved. To isolate excitatory features, we identified the squared pixel values of the filtered image that were above a pre-defined threshold. Threshold values were determined for each filtered image to equate the number of above-threshold pixels with the size of the corresponding spike-triggered mask.

Predicted suppressive features were computed by filtering with 1-SRF and applying the same threshold value used to isolate excitatory features. Since V4 neurons often exhibit little or no spontaneous activity, this method is likely to overestimate the area of the suppressive features. However, our approach provides a reasonable approximation given the intrinsic limitations on measuring inhibitory processes using extracellular recording methods and closely resembles approaches taken in previous studies (Chen et al., 2005; Rust et al., 2005).

## Results

We obtained spatial receptive fields, SRFs, and spike-triggered masks from 91 V4 neurons in two monkeys (37 in monkey P and 54 in monkey F) performing a passive fixation task. Our preliminary analysis revealed no significant differences between data from the two animals so they were combined. The spike-triggered masks calculated for a typical V4 neuron are shown in Figure 1B, along with the corresponding natural images used to estimate each mask. Across the population, response latencies (i.e., time to peak) for unmasked natural image stimuli were 90.9 ± 35.5 ms (*n* = 91). Latencies for masked image stimuli were similar (*n* = 91; 101.9 ± 33.0 ms). Based on these numbers, we used a fixed temporal integration window of 50–150 ms after stimulus onset to calculate spike-triggered masks across the entire set of V4 neurons studied here. Pixels that contributed significantly to each spike-triggered mask were identified using the bootstrap method (see Materials and Methods) and the remaining pixels (*p* > 0.01) were set to zero. We obtained a valid spike-triggered mask, with at least one statistically significant driving feature, in 95% (89/91) of V4 neurons.

**Figure 4:**
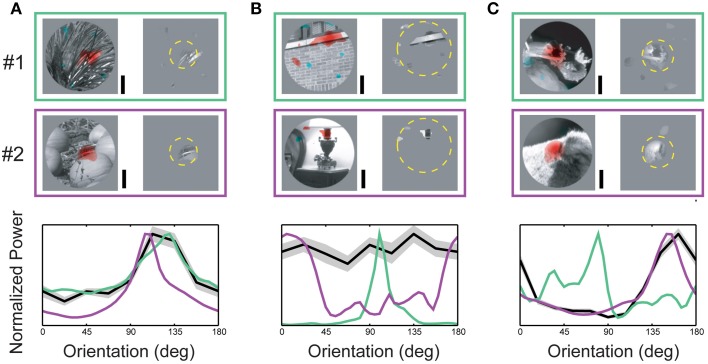
**Orientation content of driving features. (A–C)** Upper two rows (#1, #2) indicate spike-triggered masks obtained from a single neuron for two different base images. The left side of each panel shows the underlying base image with red and blue coding excitatory and suppressive driving features, respectively. The right side of each panel shows the image pixels determined to significantly contribute to neuronal responses (see Materials and Methods). Dashed yellow circle indicates RF size and position. Plots in the lower row of each panel illustrate the relationship between each neuron's orientation tuning profile (from the SRF) and the orientation power of the measured driving features in each spike-triggered mask. Green and purple curves indicate orientation power for the combined masks and images in #1 and #2, respectively; black curve indicates each neuron's orientation tuning (± std) from the SRF. **(A)** Example of a V4 neuron where conventional orientation tuning closely matches the orientation spectrum of the image features captured in the spike-triggered mask. For this neuron, the correspondence between conventional tuning and driving feature content is maintained for both base images. **(B)** V4 neuron with mismatched conventional tuning and driving feature content. **(C)** V4 neuron with a partial match between tuning and driving feature content. For this neuron, the tuning profile closely matched the driving feature orientation for one base image, but not the other. Scale bars = 0.5°.

Spike-triggered masks from three neurons representative of the overall population are illustrated in Figure 4. In all three example neurons, the spike-triggered masks were smaller than the CRF (dashed yellow circle)—the mask shown in Figure 4B was a full order of magnitude smaller than the RF. Across the population of neurons, spike-triggered masks were significantly smaller than the CRF (24.2 ± 32.1%, *p* < 0.001 Wilcoxon signed-rank test, *n* = 89). The bottom panels in Figure 4 show orientation power of the image pixels inside the spike-triggered masks (green and purple lines) compared with the neuron's orientation tuning from the SRF (black line). For the neuron in Figure 4A the orientation spectra within the masks for both images were similar and closely matched the cell's SRF-derived orientation tuning. In contrast, the orientation content in masks for the two different images in Figure 4B was almost orthogonal, while the neuron's SRF indicated little or no orientation tuning at all. Figure 4C illustrates an intermediate case, a neuron with strong SRF orientation tuning which matches the spectral content of only one of the spike-triggered masks. These examples reflect the diversity of both SRF tuning in V4 and the range of correspondence between the SRF and the spectral content within the spike-triggered masks.

**Figure 5:**
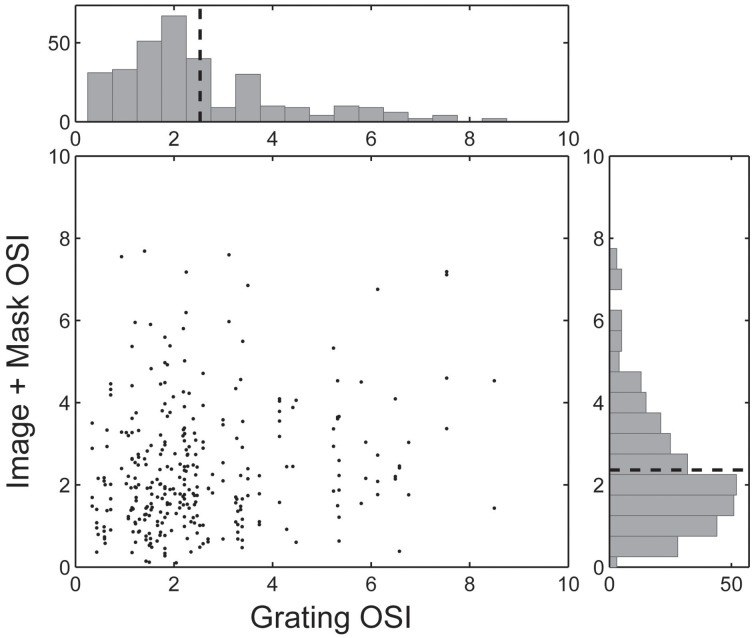
**Pair-wise comparison of the Orientation Selectivity Index (OSI) for each spike-triggered mask**. Each neuron's Grating OSI (x-axis) was calculated from the responses to the dynamic grating sequence across all spatial frequencies. Image + Mask OSIs (y-axis) were calculated by first applying each neuron's spike-triggered mask to the underlying base image and computing the dot product (i.e., similarity) between the masked image and dynamic grating stimuli. Histograms are marginal density plots and dashed lines indicate the mean OSI values across the population (*n* = 292 masks, *n* = 89 neurons).

For each neuron we characterized orientation tuning strength using the orientation selectivity index (OSI, Chen et al., 2005), which ranged from 0.09 (non-selective) to 7.81 (highly selective), with an average value of 2.07 ± 1.60 (see Figure 5). However, as illustrated in Figure 4B and summarized in the scatter plot in Figure 5, the driving features of many broadly tuned V4 neurons had narrowband orientation spectra, even though the stimulus set included many images with spatially extensive broadband texture patterns that more closely matched their broad orientation tuning profiles. For these neurons, the SRF alone was insufficient to account for image selectivity.

**Figure 6:**
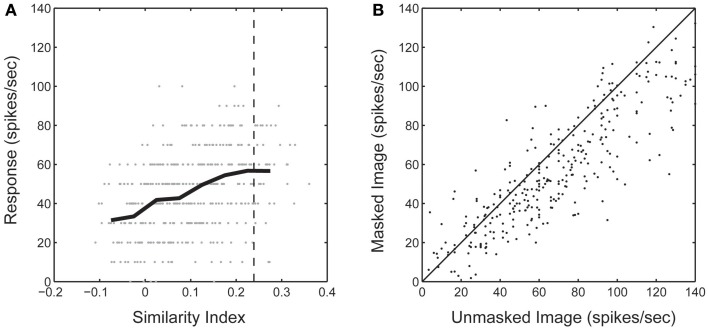
**(A)** Sorted neural response during the bubbles stimulus sequence from one neuron. Neural response is shown as a function of the similarity (dot product) between each frame of the bubbles mask stimuli and the resultant spike-triggered mask. Negative values can be achieved due to the suppressive components of the spike-triggered mask. Dashed line indicates 90th percentile threshold for this neuron. **(B)** Average response rate from the original unmasked natural image compared with the response from masked images with a similarity index in the 90th percentile.

**Figure 7:**
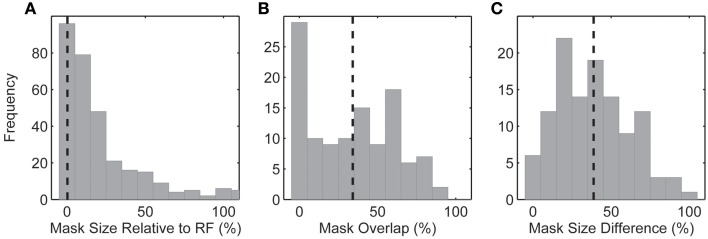
**(A)** Size of the spike-triggered mask relative to the spatial RF. **(B)** Fractional (%) overlap between the multiple spike-triggered masks derived from each neuron. Spike-triggered masks were calculated for each of the 2–6 underlying natural images. **(C)** Mask size difference between the multiple spike-triggered masks derived from each neuron. Percentage calculated relative to the largest spike-triggered mask for each neuron. Dashed lines indicate the mean of each distribution.

We wanted to confirm that mismatches between the spike-triggered mask's spectral content and corresponding SRF were not simply a general failure of the method to correctly identify driving features in natural images. To accomplish this, we computed the dot product between the spike-trigged mask and each mask in the stimulus sequence. This gave us an index of how similar each frame of the stimulus sequence was to the resulting spike-triggered mask (Figure 6A). Across the population, we found that responses to those frames of the stimulus sequence with masks most similar to the spike-triggered mask (90th percentile) were only marginally attenuated compared to the unmasked stimulus (62.0 ± 32.6 vs. 74.9 ± 38.9 spikes/s; Figure 6B). This was true even though only 32% of a masked image was visible on any given stimulus frame. These results indicate that the spike-triggered masks were effective in reliably isolating the portions of each image driving the spiking response; or stated another way, stimulus features outside the masks, but still inside the RF, made little or no contribution to the spiking response, even though they fell within the boundaries of the classical RF. Figure 7 summarizes the spatial relationship between the RF and the spike-triggered masks across the population of neurons studied and shows that masks were consistently smaller than the classical RF.

### Predicting driving features from the SRF

**Figure 8:**
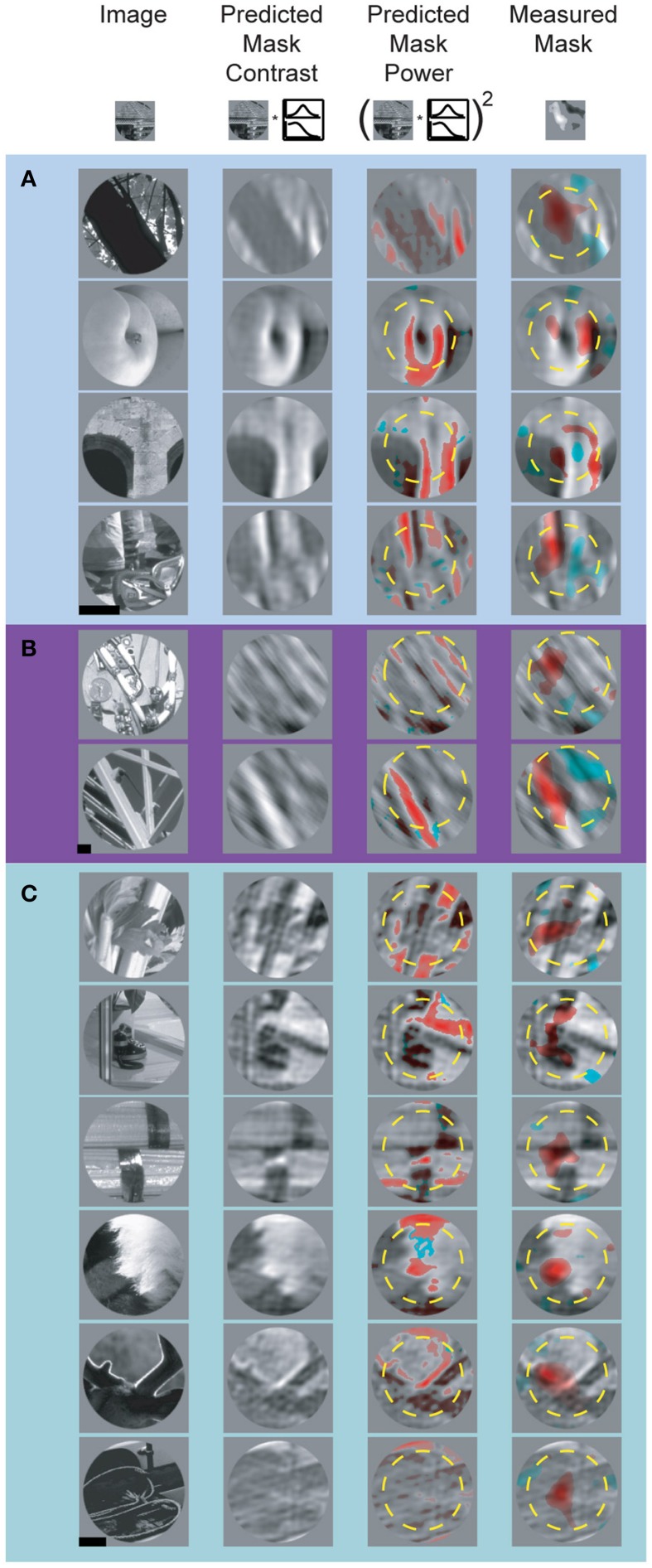
**Predicted and measured spike-triggered masks for three representative V4 neurons (A–C)**. Left-most column shows underlying base images, two middle columns show the SRF-filtered base image and image contrast, respectively (see Materials and Methods). The right-most column shows measured spike-triggered masks for the same images. In the two right hand columns, red and blue overlay indicates excitatory and suppressive feature components of the predicted and measured masks. For display purposes, when the predicted excitatory and suppressive masks overlap, only the excitatory mask is shown. Yellow dashed circles indicate RF size and position. Scale bar = 0.5°.

Accurately modeling and predicting the responses of visual neurons to arbitrary stimuli is an essential step toward a full understanding of visual cortex. Specific failures of well-articulated models can be highly informative and provide insight on how to improve said models. Our data reveal several important failures of the linear model of feature selectivity derived from the spatial and spectral tuning profiles. After confirming the spike-triggered mask robustly identified driving features in natural images, we asked to what degree spike-triggered masks could be predicted from the SRF using a V1-like quasi-linear model of selectivity (David et al., 2006). To accomplish this, we computed the predicted excitatory feature mask by filtering the unmasked natural image stimuli with the normalized SRF and applying a threshold value that equated the area of the predicted and measured spike-trigged mask (see Materials and Methods). This approach preserves features that contain spectral components within each neuron's passband. Although the “top” images used to isolate driving features were selected because they robustly increased firing rate, in many cases we observed suppressive mask regions containing pixels which caused a reduction in firing rate. To include this in our predictive model, we computed the suppressive feature masks by filtering images with 1-SRF and applying the same threshold used to isolate the excitatory features. Predicted and measured masks for representative V4 neurons are shown in Figure 8. Masks illustrated in Figures 8A,C are from cells depicted in Figures 3A,B, respectively (i.e., the spatial and spectral RFs shown in Figure 3 were used to generate the predicted masks in Figure 8). We found that in general, the SRF failed to identify the majority of suppressive features. For example, the measured masks for the neurons illustrated in Figures 8A,B include prominent suppressive regions, while little or no suppression is apparent in the SRF-predicted masks.

**Figure 9:**
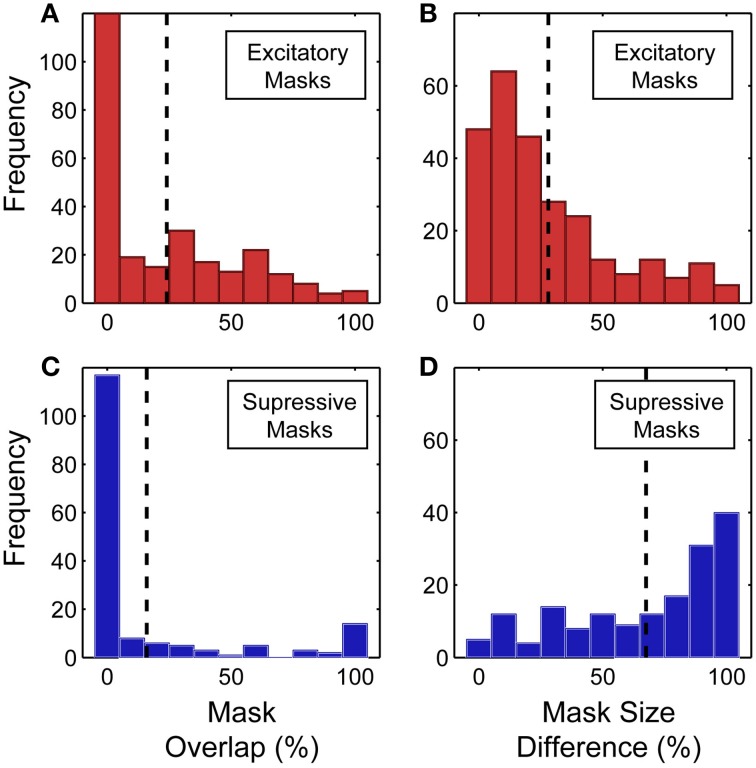
**(A,B)** Overlap between the excitatory component of the predicted and measured spike-triggered masks. Relative size difference between the excitatory component of the predicted and measured spike-triggered masks. **(C,D)** Overlap between the suppressive component of the predicted and measured spike-triggered masks. Relative size difference between the suppressive component of the predicted and measured spike-triggered masks. Percentage calculated relative to the largest of each mask pair. Dashed lines indicate the mean of each distribution.

Across the population, there was a partial overlap between the predicted and measured spike-triggered masks for both the excitatory and suppressive components (see Figure 9 for summary of mask overlap and size difference). However, we found better correspondence between the predicted and measured excitatory spike-triggered masks (mean overlap: 24.0 ± 28.1%; *n* = 265; Figure 9A) compared with the suppressive masks (mean overlap: 15.9 ± 31.8%; *n* = 164; Figure 9C), a significant difference in overlap (*p* < 0.01, sign test; *n* = 429). This indicates that the SRF, measured using traditional narrowband stimuli, fails to adequately model feature selectivity, particularly the spectrally tuned suppression found in V4.

### Broadly tuned but highly selective neurons

We found many V4 neurons that displayed broad orientation and/or spatial frequency tuning when probed with sinusoidal gratings, yet exhibited highly significant and reproducible spike-triggered masks (e.g., Figure 4B) not predicted by the SRF model. This suggests that under naturalistic viewing conditions, responses may be driven by a small subset of orientation or spatial frequency channels that are components of a larger spectral passband. This highly non-linear property is consistent with previous findings that some V4 neurons respond preferentially to feature conjunctions that occur only in spectrally complex stimuli (Kobatake and Tanaka, 1994). Dynamic gratings, while a useful, efficient and powerful stimulus, are spectrally narrowband and consequently may not adequately sample the space of feature conjunctions required to maximally drive highly selective V4 neurons. Natural images, however, are both spectrally complex and have a high probability of containing multiple features spanning a range of spatial scales (Field, 1987), which could explain why many neurons with broadly-selective SRFs were highly selective for complex feature components in the natural scene stimuli.

### Hidden suppressive tuning

Another important failure of the linear SRF model is an inability to identify suppressive features that contain orientations similar to the neuron's preferred orientation. This can be seen in Figures 8A,B, where the pixels inside the excitatory and suppressive spike-triggered masks have similar orientation content (e.g., Figure 8A: row 3 and Figure 8B: row 2). As a result, features predicted by the SRF to be excitatory can actually be either excitatory or suppressive, depending on their location within the CRF. This similarity between the excitatory and suppressive features reflects the fact that complex feature selectivity in V4 is neither uniform nor exclusively excitatory within the CRF. The narrow-band SRF, by its very design, is unable to model this overlapping, differentially tuned aspect of feature selectivity.

### Excitatory features outside the CRF

We found more than 80% (73/91) of the neurons studied had spike-triggered masks with significant excitatory components lying outside the CRF. This is surprising, since the CRF is defined (and measured here) as the region where isolated stimuli can elicit action potentials (Allman et al., 1985). While today the definition of the CRF is a matter of some debate, even in area V1, studies in both striate and extrastriate areas have generally found largely suppressive effects for stimuli outside the CRF (Li and Li, 1994; Walker et al., 2000).

### Translation invariance

**Figure 10:**
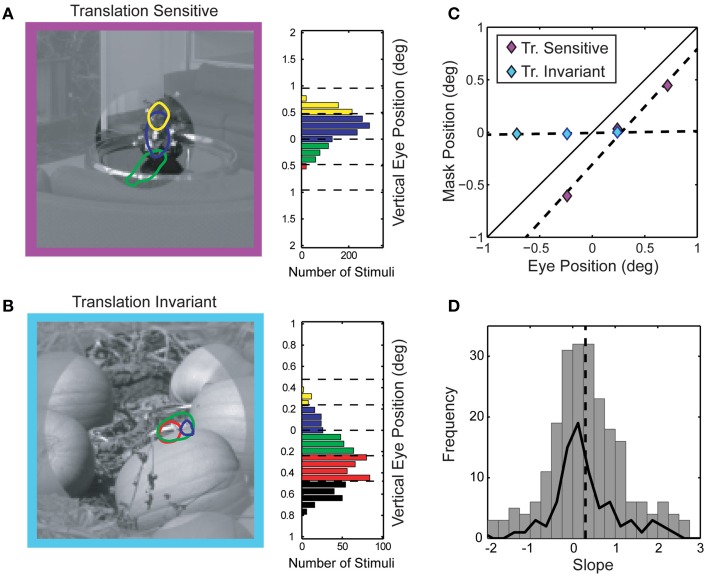
**Position sensitivity of spike-triggered masks. (A)** Gaze-angle contingent spike-triggered masks from a position sensitive V4 neuron. Colors (masks and histograms) denote modal vertical eye position and correspond to the eye position histogram on the right. The portion of image shown in low contrast was not visible during experiments and is shown here for display purposes only. The linear relationship between mask position and gaze angle is diagnostic for positive sensitivity (see text for explanation). **(B)** Position insensitive V4 neuron. For this neuron, the spike-triggered masks do not shift with eye position but rather remain fixed in head-centered coordinates. **(C)** Center of mass (vertical component only) of each of the spike-triggered masks from **(A)** and **(B)** plotted as a function of vertical eye position. **(D)** Distribution of position sensitivity slopes across the population of neurons. Average slope of 0.30 ± 1.61 is indicated by the dashed line (*n* = 206 spike-triggered masks). Solid line indicates the distribution of position sensitivity slopes for each neuron, averaging the slope across all spike-triggered masks for that neuron (*n* = 84 neurons). The average slope was significantly less than one, indicating a degree of position invariance across the population of V4 neurons studied.

Perhaps the most interesting failure of the linear model we observed in this study is an intermediate form of translation invariance that could provide V4 cells some degree of insensitivity to small eye movements. Translation invariance, where visual selectivity remains constant over a wide range of spatial positions, is an emergent property of the ascending ventral stream (Ungerleider and Mishkin, 1982; Desimone et al., 1984; Op De Beeck and Vogels, 2000; Pasupathy and Connor, 2002) and has been previously described in V4 (Connor et al., 1996, 1997; Rust and Dicarlo, 2010), but not in the context of eye movements. Computational models of object recognition posit that translation invariance is a critical and necessary property of robust object recognition (Riesenhuber and Poggio, 2002). Phase invariance, such as that seen in complex cells in V1, yields translation invariant selectivity for narrowband stimuli which is likely preserved in the ascending visual pathway. However, translation insensitive tuning for spectrally complex features, which requires preservation of relative spatial phase relations between spatial frequency channels, is more difficult to reconcile with a simple feed-forward model of V4. To characterize translation invariance in V4 natural image responses, we re-compute spike-triggered masks using gaze angle as a conditioning variable. Since monkeys were allowed to make fixational eye movements of up to 1°, there were periods within each trial where the gaze angle was not directed exactly at the fixation target. This behavior led to small but measurable shifts in the position of the stimulus sequence within the RF. We took advantage of these small fixational eye movements and explored how spike-triggered masks were affected by the exact position of the stimulus relative to the RF. Here, we focused exclusively on vertical eye movements (both monkeys in this study had a tendency to make larger and more frequent vertical rather than horizontal fixational eye movements). We computed modal vertical eye position for each stimulus frame and assigned both the stimulus frame and corresponding response to one of four bins (see Figure 10). We then computed spike-triggered masks from stimulus frames assigned to each bin separately. Figures 10A,B shows the conditional spike-triggered masks typical of translation sensitive (Figure 10A) and insensitive (Figure 10B) neurons. Mask centers (centroids) were plotted against eye position (Figure 10C) and fit with linear regression. The slope of the best-fit line indicates degree of translation invariance: one for linear, translation sensitive neurons (purple) and near zero for translation invariant neurons (light blue).

Assessing the statistical significant of this translation metric for each neuron was difficult, due to the limitations of the data; the linear regression fitting process contained at most four points for each spike-triggered mask. Therefore, we assessed the distribution of slope values across the population to determine if the mean was significantly different from one (translation sensitive). Indeed, the population of V4 neurons was significantly translation insensitive (Figure 10D; *p* < 0.01, one-tailed *t*-test, *n* = 260), with an average slope closer to zero (0.30 ± 1.61). Since we estimated spike-triggered masks using 2–6 base images for each neuron, we also calculated position insensitivity based on the average slope for each neuron and found a similar result (average slope = 0.27 ± 0.92, *p* << 0.01 one-tailed *t*-test, *n* = 84). Interestingly, the mean of this distribution was also significantly greater than zero (*p* < 0.01 one-tailed *t*-test, *n* = 84), indicating some systematic relationship between eye position and the spike-triggered masks. Likewise, while the fixational eye movements described here were of a similar magnitude as the average RF size (~1°), we found no evidence of a link between RF size and the degree of translational invariance (correlation coefficient between RF size and average slope = 0.142, *p* = 0.20). Thus, the a substantial portion of V4 neurons show some measure of translation invariant selectivity for driving features within their RF on the order of one degree (average RF radius = 0.98 ± 1.10°, *n* = 90).

## Discussion

In this study we used neurophysiological responses to partially masked natural scene stimuli to explore the origins of feature selectivity in spectrally complex natural scene stimuli in area V4 of the primate. Our results indicate feature selectivity in V4 is highly non-linear, and while the quasi-linear SRF model can predict a substantial fraction of V4 response variance under some conditions, it is not sufficient to fully model selectivity for spectrally complex stimuli (Oleskiw et al., 2014). Importantly, the pattern of differences between the SRF model and spike-triggered masks revealed at least four key failures of the linear model, most significantly tuned suppression and a complex form of translation invariance that appears to make feature selectivity in some V4 neurons robust to fixational eye movements. We also found that many V4 neurons whose SRFs indicated broad or even non-selective spectral tuning were in fact highly selective for specific visual features embedded in natural images. These results support the idea of a continuum of tuning properties in V4, ranging from “V1-like” linear cells, to highly selective cells with highly non-linear preferences for specific conjunctions of spectral features (Hegde and Van Essen, 2007).

Characterizing the feature selectivity of high-level visual neurons can be a difficult proposition. In early visual areas, system identification methods that use Gaussian white noise, dynamic gratings or other uncorrelated stimuli offer a principled, and in some sense optimal, way to obtain a comprehensive description of first-order feature selectivity. However, in later visual areas, where neurons are selective for feature conjunctions and exhibit other non-linear properties (Pasupathy and Connor, 2002), these unbiased methods are unlikely to sample the appropriate image subspace densely enough to build accurate general models of selectivity. One way to address this problem is to use stimuli derived from theoretical models of object recognition. Previous studies have probed visual neurons with stimuli that span a particular complete and over-complete basis set, including non-Cartesian gratings (Gallant et al., 1993), Walsh patterns (Richmond et al., 1987) or Hermite functions (Victor et al., 2006). Other work has used fully parameterized, but non-orthonormal, stimuli that span a perceptually defined shape space (Pasupathy and Connor, 2002). This approach consistently reveals that neurons in V4 (and other downstream ventral areas) can be highly tuned along any number of complex image dimensions. However, it has been difficult to relate results from studies using one set or class of stimuli to those obtained using others, since their neural representations are not fully understood. Another alternative is to use naturalistic stimuli to characterize visual selectivity (David et al., 2004, 2006; Touryan et al., 2005; Willmore et al., 2010), as we have done here. While this approach can still fail to span a sufficient fraction of the image space, it is highly likely that evolutionary pressure has guided vertebrate visual systems toward selectivity for the features and feature conjunctions commonly found in natural scenes (a subspace of all possible images of a given dimensionality). However, the complexity of naturalistic stimuli, along with their highly non-uniform spectral properties (Field, 1987), makes estimating linear selectivity, let alone higher order non-linear tuning properties, a formidable methodological challenge (Theunissen et al., 2001).

The bubble mask approach represents a compromise between these two extremes. By using naturalistic stimuli, we are able to reliably find stimuli that drive virtually all V4 neurons, suggesting we have identified the right image subspace. The uniform, random positioning of the Gaussian windows means that distribution of mask positions is unbiased (i.e., white) and therefore amenable to robust system identification techniques. While the bubbles approach used here is not a complete system identification method (Gosselin and Schyns, 2004; Murray and Gold, 2004), it offers an efficient and robust approach for identifying the key image components that modulate spiking activity in individual V4 neurons. Understanding the significance of those components requires a second step of analysis, in this case hypothesis testing to compare measured image components with predictions from the linear SRF model. When used this way, the bubble technique allowed us to identify and characterize non-linear feature selectivity with far less data than would have been required using system identification methods like spike-triggered covariance and other higher-order kernel estimation techniques (Marmarelis and Marmarelis, 1978; Schwartz et al., 2006).

Given the diversity of tuning properties and high degree of selectivity in V4, it should come as no surprise that non-linearities are prevalent in this visual area. Nonetheless, our findings indicate the linear model can still be a useful starting point from which to explore complex feature selectivity. It is clear from our data that not all V4 neurons are simply excited by visual features that fall within their spectral pass band and CRF. In this study, we have shown that near-optimal spectral features (based on first-order tuning) can be either excitatory or suppressive, both within and outside the CRF. In addition, we have shown that a number of V4 neurons exhibit a form of translation invariance that potentially makes complex feature selectivity immune to small eye movements. Explicit incorporation of these non-linearities into physiologically motivated models of visual processing and object recognition will improve our understanding of neural coding in extrastriate cortex.

### Conflict of interest statement

The authors declare that the research was conducted in the absence of any commercial or financial relationships that could be construed as a potential conflict of interest.

## References

[B1] Allman J. Miezin F. Mcguiness E. (1985). Stimulus-specific responses from beyond the classical receptive field: neurophysiological mechanisms for local-global comparisons on visual neurons. Annu. Rev. Neurosci. 8, 407–430. 10.1146/annurev.ne.08.030185.002203 3885829

[B2] Bushnell B. N. Pasupathy A. (2012). Shape encoding consistency across colors in primate V4. J. Neurophysiol. 108, 1299–1308. 10.1152/jn.01063.2011 22673324 PMC3544963

[B3] Chen G. Dan Y. Li C. Y. (2005). Stimulation of non-classical receptive field enhances orientation selectivity in the cat. J. Physiol. 564, 233–243. 10.1113/jphysiol.2004.080051 15677690 PMC1456041

[B4] Chichilnisky E. J. (2001). A simple white noise analysis of neuronal light responses. Network 12, 199–213. 10.1080/713663221 11405422

[B5] Connor C. E. Gallant J. L. Preddie D. C. Van Essen D. C. (1996). Responses in area V4 depend on the spatial relationship between stimulus and attention. J. Neurophys. 75, 1306–1308. 8867139 10.1152/jn.1996.75.3.1306

[B6] Connor C. E. Preddie D. C. Gallant J. L. Van Essen D. C. (1997). Spatial attention effects in macaque area V4. J. Neurosci. 17, 3201–3214. 9096154 10.1523/JNEUROSCI.17-09-03201.1997 PMC6573654

[B7] David S. V. Hayden B. Y. Gallant J. L. (2006). Spectral receptive field properties explain shape selectivity in area V4. J. Neurophysiol. 96, 3492–3505. 10.1152/jn.00575.2006 16987926

[B8] David S. V. Vinje W. E. Gallant J. L. (2004). Natural stimulus statistics alter the receptive field structure of v1 neurons. J. Neurosci. 24, 6991–7006. 10.1523/JNEUROSCI.1422-04.2004 15295035 PMC6729594

[B9] Deangelis G. C. Ohzawa I. Freeman R. D. (1993). Spatiotemporal organization of simple-cell receptive fields in the cat's striate cortex. II. Linearity of temporal and spatial summation. J. Neurophysiol. 69, 1118–1135. 8492152 10.1152/jn.1993.69.4.1118

[B10] Desimone R. Albright T. D. Gross C. G. Bruce C. (1984). Stimulus-selective properties of inferior temporal neurons in the macaque. J. Physiol. 357, 219–240. 6470767 10.1523/JNEUROSCI.04-08-02051.1984 PMC6564959

[B11] Desimone R. Schein S. J. (1987). Visual properties of neurons in area V4 of the macaque: sensitivity to stimulus form. J. Neurophys. 57, 835–868. 3559704 10.1152/jn.1987.57.3.835

[B12] Efron B. Tibshirani R. (1993). An Introduction to the Bootstrap. New York, NY: Chapman & Hall 10.1007/978-1-4899-4541-9

[B13] Felsen G. Dan Y. (2005). A natural approach to studying vision. Nat. Neurosci. 8, 1643–1646. 10.1038/nn1608 16306891

[B13a] Felsen G. Touryan J. Han F. Dan Y. (2005). Cortical sensitivity to visual features in natural scenes. PLoS Biol. 3:e342. 10.1371/journal.pbio.0030342 16171408 PMC1233414

[B14] Field D. J. (1987). Relations between the statistics of natural images and the response properties of cortical cells. J. Opt. Soc. Am. A 4, 2379–2394. 10.1364/JOSAA.4.002379 3430225

[B15] Gallant J. L. Braun J. Van Essen D. C. (1993). Selectivity for polar, hyperbolic, and Cartesian gratings in macaque visual cortex. Science 259, 100–103. 10.1126/science.8418487 8418487

[B16] Gosselin F. Schyns P. G. (2001). Bubbles: a technique to reveal the use of information in recognition tasks. Vision Res. 41, 2261–2271. 10.1016/S0042-6989(01)00097-9 11448718

[B17] Gosselin F. Schyns P. G. (2004). No troubles with bubbles: a reply to Murray and Gold. Vision Res. 44, 471–477. discussion 479–482. 10.1016/j.visres.2003.10.007 14680772

[B18] Hanazawa A. Komatsu H. (2001). Influence of the direction of elemental luminance gradients on the responses of V4 cells to textured surfaces. J. Neurosci. 21, 4490–4497. 11404436 10.1523/JNEUROSCI.21-12-04490.2001 PMC6762768

[B19] Heeger D. J. (1992). Normalization of cell responses in cat striate cortex. Vis. Neurosci. 9, 181–197. 10.1017/S0952523800009640 1504027

[B20] Hegde J. Van Essen D. C. (2007). A comparative study of shape representation in macaque visual areas V2 and V4. Cereb. Cortex. 17, 1100–1116. 10.1093/cercor/bhl020 16785255

[B21] Hinkle D. A. Connor C. E. (2001). Disparity tuning in Macaque area V4. Neuroreport 12, 365–369. 10.1097/00001756-200102120-00036 11209951

[B22] Hinkle D. A. Connor C. E. (2002). Three-dimensional orientation tuning in macaque area V4. Nat. Neurosci. 5, 665–670. 10.1038/nn875 12068303

[B23] Jones J. P. Palmer L. A. (1987). The two-dimensional spatial structure of simple receptive fields in cat striate cortex. J. Neurophysiol. 58, 1187–1211. 3437330 10.1152/jn.1987.58.6.1187

[B24] Kobatake E. Tanaka K. (1994). Neuronal selectivities to complex object features in the ventral visual pathway of the macaque cerebral cortex. J. Neurophys. 71, 856–867. 8201425 10.1152/jn.1994.71.3.856

[B25] Li C. Y. Li W. (1994). Extensive integration field beyond the classical receptive field of cat's striate cortical neurons–classification and tuning properties. Vision Res. 34, 2337–2355. 10.1016/0042-6989(94)90280-1 7975275

[B26] Marmarelis P. Z. Marmarelis V. Z. (1978). Analysis of Physiological Systems: The White Noise Approach. New York, NY: Plenum 10.1007/978-1-4613-3970-0

[B27] Mazer J. A. Vinje W. E. Mcdermott J. Schiller P. H. Gallant J. L. (2002). Spatial frequency and orientation tuning dynamics in area V1. Proc. Natl. Acad. Sci. U.S.A. 99, 1645–1650. 10.1073/pnas.022638499 11818532 PMC122244

[B28] Murray R. F. Gold J. M. (2004). Troubles with bubbles. Vision Res. 44, 461–470. 10.1016/j.visres.2003.10.006 14680771

[B29] Nielsen K. J. Logothetis N. K. Rainer G. (2006). Dissociation between local field potentials and spiking activity in macaque inferior temporal cortex reveals diagnosticity-based encoding of complex objects. J. Neurosci. 26, 9639–9645. 10.1523/JNEUROSCI.2273-06.2006 16988034 PMC6674446

[B30] Nielsen K. J. Logothetis N. K. Rainer G. (2008). Object features used by humans and monkeys to identify rotated shapes. J. Vis. 8, 9.1–9.15. 10.1167/8.2.9 18318635

[B31] Ohzawa I. Sclar G. Freeman R. D. (1982). Contrast gain control in the cat visual cortex. Nature 298, 266–268. 10.1038/298266a0 7088176

[B32] Oleskiw T. D. Pasupathy A. Bair W. (2014). Spectral receptive fields do not explain tuning for boundary curvature in V4. J. Neurophysiol. 112, 2114–2122. 10.1152/jn.00250.2014 25057148 PMC4274922

[B33] Op De Beeck H. Vogels R. (2000). Spatial sensitivity of macaque inferior temporal neurons. J. Comp. Neurol. 426, 505–518. 10.1002/1096-9861(20001030)426:4<505::AID-CNE1>3.0.CO;2-M 11027395

[B34] Pasupathy A. Connor C. E. (2001). Shape representation in area V4: position-specific tuning for boundary conformation. J. Neurophysiol. 86, 2505–2519. 11698538 10.1152/jn.2001.86.5.2505

[B35] Pasupathy A. Connor C. E. (2002). Population coding of shape in area V4. Nat. Neurosci. 5, 1332–1338. 10.1038/972 12426571

[B36] Richmond B. J. Optican L. M. Podell M. Spitzer H. (1987). Temporal encoding of two-dimensional patterns by single units in primate inferior temporal cortex. I. Response characteristics. J. Neurophys. 57, 132–146. 10.1152/jn.1987.57.1.132 3559668

[B37] Riesenhuber M. Poggio T. (2002). Neural mechanisms of object recognition. Curr. Opin. Neurobiol. 12, 162–168. 10.1016/S0959-4388(02)00304-5 12015232

[B38] Ringach D. L. Hawken M. J. Shapley R. (1997). Dynamics of orientation tuning in macaque primary visual cortex. Nature 387, 281–284. 10.1038/387281a0 9153392

[B39] Rust N. C. Dicarlo J. J. (2010). Selectivity and tolerance (“invariance”) both increase as visual information propagates from cortical area V4 to IT. J. Neurosci. 30, 12978–12995. 10.1523/JNEUROSCI.0179-10.2010 20881116 PMC2975390

[B40] Rust N. C. Movshon J. A. (2005). In praise of artifice. Nat. Neurosci. 8, 1647–1650. 10.1038/nn1606 16306892

[B41] Rust N. C. Schwartz O. Movshon J. A. Simoncelli E. P. (2005). Spatiotemporal elements of macaque v1 receptive fields. Neuron 46, 945–956. 10.1016/j.neuron.2005.05.021 15953422

[B42] Schwartz O. Pillow J. W. Rust N. C. Simoncelli E. P. (2006). Spike-triggered neural characterization. J. Vis. 6, 484–507. 10.1167/6.4.13 16889482

[B43] Theunissen F. E. David S. V. Singh N. C. Hsu A. Vinje W. E. Gallant J. L. (2001). Estimating spatial temporal receptive fields of auditory and visual neurons from their responses to natural stimuli. Network 12, 289–316. 10.1088/0954-898X/12/3/304 11563531

[B44] Touryan J. Felsen G. Dan Y. (2005). Spatial structure of complex cell receptive fields measured with natural images. Neuron 45, 781–791. 10.1016/j.neuron.2005.01.029 15748852

[B45] Touryan J. Lau B. Dan Y. (2002). Isolation of relevant visual features from random stimuli for cortical complex cells. J. Neurosci. 22, 10811–10818. 12486174 10.1523/JNEUROSCI.22-24-10811.2002 PMC6758424

[B46] Ungerleider L. G. Mishkin M. (1982). Two cortical visual systems, in Analysis of Visual Behavior, eds Ingle D. G. Goodale M. A. Mansfield R. J. Q. (Cambridge, MA: MIT Press), 549–586.

[B47] Victor J. D. Mechler F. Repucci M. A. Purpura K. P. Sharpee T. (2006). Responses of V1 neurons to two-dimensional hermite functions. J. Neurophysiol. 95, 379–400. 10.1152/jn.00498.2005 16148274 PMC2927229

[B48] Walker G. A. Ohzawa I. Freeman R. D. (2000). Suppression outside the classical cortical receptive field. Vis. Neurosci. 17, 369–379. 10.1017/S0952523800173055 10910105

[B49] Willmore B. D. Prenger R. J. Gallant J. L. (2010). Neural representation of natural images in visual area V2. J. Neurosci. 30, 2102–2114. 10.1523/JNEUROSCI.4099-09.2010 20147538 PMC2994536

[B50] Wu M. C. David S. V. Gallant J. L. (2006). Complete functional characterization of sensory neurons by system identification. Annu. Rev. Neurosci. 29, 477–505. 10.1146/annurev.neuro.29.051605.113024 16776594

[B51] Zeki S. (1980). The representation of colours in the cerebral cortex. Nature 284, 412–418. 10.1038/284412a0 6767195

