# Supplementary material for: Linear and non-linear properties of feature selectivity in V4 neurons
Source: Front Syst Neurosci. 2015 May 27;9:82. doi: 10.3389/fnsys.2015.00082 (PMC4444755; doi:10.3389/fnsys.2015.00082)
Supplement: Supplementary file 1 [file Image1.PDF]

Supplementary Material

Linear and Nonlinear Properties of Feature Selectivity in V4 Neurons

Jon Touryan[1,3,§] and James A. Mazer[1,2,*]

1. Department of Neurobiology, Yale School of Medicine, New Haven, CT 06520

2. Department of Psychology, Yale University, New Haven, CT 06520

3. Human Research and Engineering Directorate, U.S. Army Research Laboratory, Aberdeen Proving Ground, MD 21005

*** Correspondence:** James A. Mazer, PhD, Department of Neurobiology, Yale School of Medicine, New Haven, CT 06520-8001; e-mail: james.mazer@yale.edu

1. V4 Neuronal Population

V4 was targeted using stereotaxic coordinates and skull morphology and subsequently confirmed based on physiological properties of recorded cells (*i.e.*, neuronal response latency, receptive field size and visual field eccentricity). Cells were sampled at all depths and isolated based on standard clustering parameters (peak amplitude, spike width, etc). Figure S1 shows RF properties of the population of neurons (n = 91) from both animals.

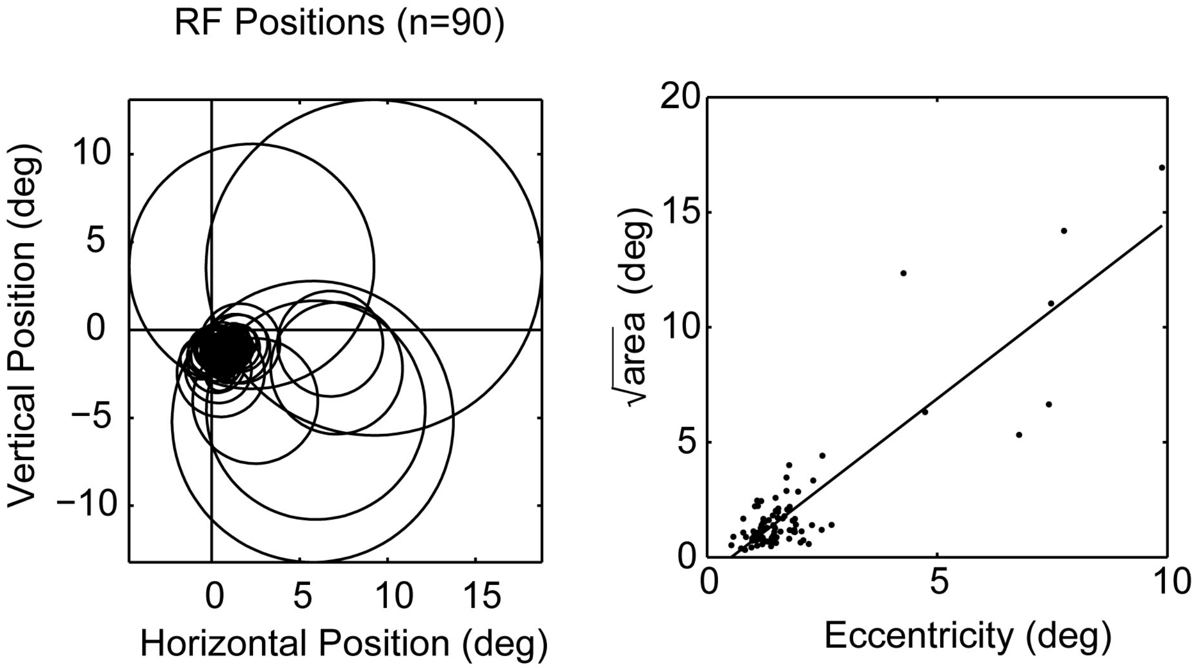

Supplementary Figure 1: RF size and position of the V4 population. The spatial RFs were mapped using high contrast black and white probe stimuli flashed in randomized order on an invisible grid at 5-10 Hz. RF location and size (radius) were determined by fitting the half-maximal iso-response contour of the spike-triggered average with a circle.
